# Pharmacological Inhibition of Monoacylglycerol O-Acyltransferase 2 Improves Hyperlipidemia, Obesity, and Diabetes by Change in Intestinal Fat Utilization

**DOI:** 10.1371/journal.pone.0150976

**Published:** 2016-03-03

**Authors:** Kazumi Take, Taisuke Mochida, Toshiyuki Maki, Yoshinori Satomi, Megumi Hirayama, Masanori Nakakariya, Nobuyuki Amano, Ryutaro Adachi, Kenjiro Sato, Tomoyuki Kitazaki, Shiro Takekawa

**Affiliations:** 1 Cardiovascular and Metabolic Drug Discovery Unit, Takeda Pharmaceutical Company Limited, Fujisawa, Japan; 2 Integrated Technology Research Laboratories, Takeda Pharmaceutical Company Limited, Fujisawa, Japan; 3 DMPK Research Laboratories, Takeda Pharmaceutical Company Limited, Fujisawa, Japan; 4 Biomolecular Research Laboratories, Takeda Pharmaceutical Company Limited, Fujisawa, Japan; Showa University School of Pharmacy, JAPAN

## Abstract

Monoacylglycerol O-acyltransferase 2 (MGAT2) catalyzes the synthesis of diacylglycerol (DG), a triacylglycerol precursor and potential peripheral target for novel anti-obesity therapeutics. High-throughput screening identified lead compounds with MGAT2 inhibitory activity. Through structural modification, a potent, selective, and orally bioavailable MGAT2 inhibitor, compound A (compA), was discovered. CompA dose-dependently inhibited postprandial increases in plasma triglyceride (TG) levels. Metabolic flux analysis revealed that compA inhibited triglyceride/diacylglycerol resynthesis in the small intestine and increased free fatty acid and acyl-carnitine with shorter acyl chains than originally labelled fatty acid. CompA decreased high-fat diet (HFD) intake in C57BL/6J mice. MGAT2-null mice showed a similar phenotype as compA-treated mice and compA did not suppress a food intake in MGAT2 KO mice, indicating that the anorectic effects were dependent on MGAT2 inhibition. Chronic administration of compA significantly prevented body weight gain and fat accumulation in mice fed HFD. MGAT2 inhibition by CompA under severe diabetes ameliorated hyperglycemia and fatty liver in HFD-streptozotocin (STZ)-treated mice. Homeostatic model assessments (HOMA-IR) revealed that compA treatment significantly improved insulin sensitivity. The proximal half of the small intestine displayed weight gain following compA treatment. A similar phenomenon has been observed in Roux-en-Y gastric bypass-treated animals and some studies have reported that this intestinal remodeling is essential to the anti-diabetic effects of bariatric surgery. These results clearly demonstrated that MGAT2 inhibition improved dyslipidemia, obesity, and diabetes, suggesting that compA is an effective therapeutic for obesity-related metabolic disorders.

## Introduction

Obesity is a major risk factor for type 2 diabetes and cardiovascular disease and is associated with an increase in energy intake relative to energy expenditure [1, 2]. Postprandial dyslipidemia in response to overfeeding with a high-fat diet (HFD) increases fat accumulation, primarily in adipose tissue, and results in obesity [3]. Excess blood lipid levels cause triglyceride (TG) deposition in the skeletal muscle, liver, and pancreas [4]. Ectopic fat storage is closely linked to systemic lipotoxicity, a critical mediator in decreased energy expenditure, insulin resistance, and impaired insulin secretion [5, 6]. TG biosynthesis occurs via 2 major pathways: the monoacylglycerol (MG) pathway and glycerol 3-phosphate pathway [7–9]. In the small intestinal mucosa, the MG pathway accounts for 70%–80% of postprandial TG synthesis [10], with subsequent incorporation of resynthesized TGs into chylomicrons for secretion into the blood and transport to peripheral tissues.

Monoacylglycerol O-acyltransferase (MGAT) catalyzes the formation of diacylglycerol (DG), a TG and phospholipid precursor, from 2-monoacylglycerol (2-MG) and fatty acyl-CoA [11]. There are three reported MGAT isoforms in human and rodent genomes [12–15]: MGAT1, mainly expressed in the stomach and kidney but not the small intestine [12], and MGAT2 (rodents and humans) and MGAT3 (humans only), both highly expressed in the small intestine [13–16].

MGAT2 mediates the rate-limiting step in intestinal TG absorption, and MGAT2-null mice have demonstrated that MGAT2 plays an important role in systemic lipid and glucose metabolism [11, 17, 18]. Mice lacking MGAT2 are protected from obesity and insulin resistance induced by HFD [18, 19]. These mice exhibit increased energy expenditure, suggesting that MGAT2 also influences systemic fat utilization [18, 19]. Therefore, MGAT2 is considered a promising pharmacological target for treating obesity and its associated diseases.

Here we present the first pharmacological profiling of compound A (compA), a novel and orally active inhibitor of MGAT2 enzymatic activity (IC_50_ = 7.8 and 2.4 nmol/L for human and mouse MGAT2, respectively) with a good pharmacokinetic profile. Our results support MGAT2 activity inhibition as a potential therapeutic strategy to counteract human obesity, diabetes, and comorbidities related to abnormal lipid metabolism.

## Methods

### Materials

**Fig 1:**
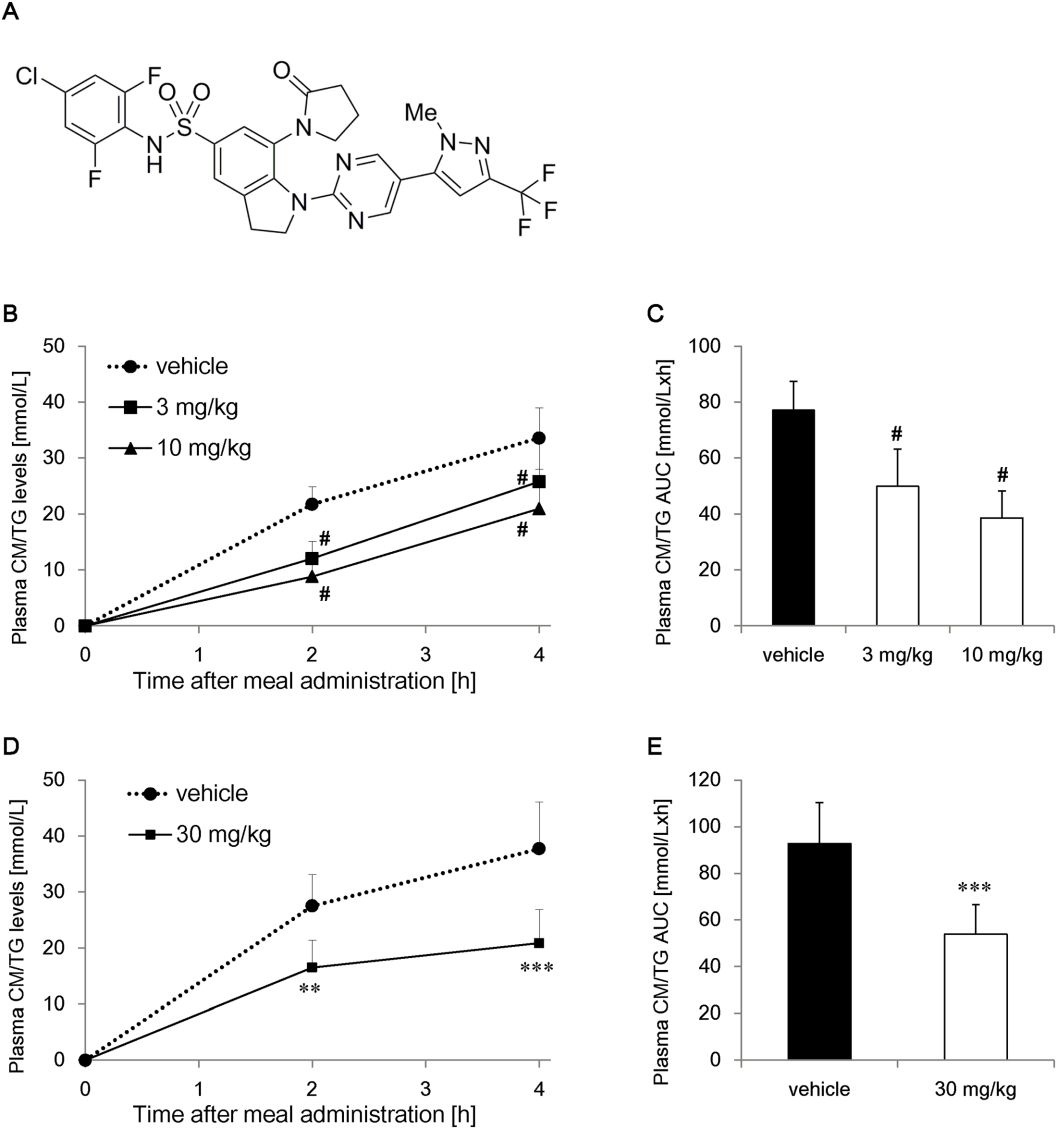
Effect of compound A (compA) on postprandial triglyceride (TG) excursion. Fasted C57BL/6J mice were given a liquid meal orally with intraperitoneal injection of Pluronic F-127 to inhibit plasma TG lipolysis. (A) Structure of compA. Plasma samples were collected at 0, 2, and 4 h after oral gavage of a liquid meal. (B) Time course of changes in plasma chylomicron TG (CM/TG) levels and (C) postprandial TG excursion of 3 or 10 mg/kg compA at 6 h after dosing. (D) Time course of changes in plasma TG levels and (E) postprandial TG excursion of 30 mg/kg compA at 16 h after dosing. n = 6 (B, C), and n = 7 (D, E). #: *P* < 0.025 vs. vehicle group by one-tailed Williams’ test. **: *P* < 0.01, ***: *P* < 0.001 vs. vehicle group by Student’s t-test.

Structure of compA was shown in Fig 1A. Synthesis of compA was reported previously [20]. This compound exhibited selectivity (greater than 30,000-fold) against related acyltransferases (MGAT3, DGAT1, DGAT2, and ACAT1) [20]. Glycerol-labeled MG (2-oleyl-[1, 1, 2, 3, 3 d_5_]-glycerol) and fatty acid-labeled MG (2-[17, 17, 18, 18, 18 d_5_]-oleoylglycerol) were purchased from CURACHEM (Gyeonggi-do, Korea). Organic solvents were purchased from WAKO (Osaka, Japan).

### Ethics Statement

The care and use of the animals and the experimental protocols used in this research were approved by the Experimental Animal Care and Use Committee of Takeda Pharmaceutical Company Limited, and the Guide for the Care and Use of Laboratory. Animals were maintained throughout the study (Institute of Laboratory Animal Resources, National Academic Press 1996; NIH publication number 85–23, revised 1996).

### Animals

Male C57BL/6J mice were obtained from CLEA Japan, Inc. (Tokyo, Japan) or Charles River Laboratories, Inc. (Kanagawa, Japan). MGAT2 knockout (KO) mice were obtained from the Jackson Laboratories (ME, USA). HFDs containing 45% (D12451) and 60% fat (D12492) were obtained from Research Diets, Inc. (NJ, USA). All mice were maintained on a normal chow (NC) (CE-2, CLEA Japan, Inc., Tokyo, Japan) or HFD under a 12-h light: dark cycle. The condition of animals were monitored every weekday by our clinical veterinarian in accordance with AAALAC guideline. Any animals did not become sick or died prior to the experimental endpoint.

### Measurement of plasma parameters and blood glycated hemoglobin levels

Blood samples were collected from the tail vein without anesthesia. Plasma glucose, TG, cholesterol and non-esterified fatty acid (NEFA) levels were measured with a 7180 autoanalyser (Hitachi High-Technologies Co., Tokyo, Japan). Blood glycated hemoglobin (GHb) levels were measured with automated GHb analysers (HLC-723G8, Tosoh, Tokyo, Japan). Plasma insulin levels were measured using a mouse insulin ELISA kit (Shibayagi, Gunma, Japan).

### Oral meal tolerance test (MTT)

Overnight-fasted mice underwent MTT in the morning. First, they were orally administered vehicle (0.5% methylcellulose solution) or compA suspended in 0.5% methylcellulose. Six or 16 h after dosing, they were intraperitoneally injected 500 mg/kg Pluronic F-127 (BASF, Ludwigshafen, Germany) to inhibit plasma TG hydrolysis by lipoprotein lipase (LPL). Thirty minutes after injection, the mice were given an oral liquid meal (10 mL/kg) comprising an admixture of corn oil and Ensure-H (3:17 v/v) (Abbott Japan Co., Ltd., Tokyo, Japan). Blood samples were collected at 0, 2 and 4 h after oral gavage of the liquid meal. Area under the curve (AUC) of chylomicron TG (CM/TG), which is synthesised from dietary fat in the small intestine, was calculated by subtracting plasma TG levels of a liquid meal-untreated group from plasma TG levels of each treated group.

### Tracer-based metabolic flux analysis of intestinal lipids

Fasted mice were orally administered vehicle or 30 mg/kg compA suspension. Two hours after dosing, they were given an oral liquid meal (10 mL/kg, prepared as described above) containing ^2^H-labeled oleoylglycerol (20 mg/mL) as a tracer. Thirty minutes after the meal, the mice were euthanized by intraperitoneal injection of 40 mg/kg pentobarbital and the small intestine was collected. The intestinal mucosa was separated by scraping the inner side of the small intestine, homogenised in 9 volumes of isopropanol, and then centrifuged. The supernatant was used directly for LC/MS (Agilent Technologies Inc., CA, USA) of the labelled lipid.

### Measurement of short-term food intake after overnight fasting

After overnight fasting, food was given for 2 h. Nine-week-old male C57BL/6J mice were given 45% HFD 16 h after dosing with compA, and the NC or 45% HFD for 30 min after dosing. Eighteen-week-old male MGAT2 KO mice or wild-type (WT) littermates were given 45% HFD for 30 min after dosing.

### Chronic dosing study in HFD-fed mice

Nine-week-old male C57BL/6J mice fed 60% HFD were dosed orally with vehicle, 30 mg/kg compA or 10 mg/kg sibutramine (Enzo Life Sciences, NY, USA) for 37 days. Body weight (BW) and food intake were measured twice a week. Whole-body composition analysis was conducted using a quantitative magnetic resonance method without anesthesia (EchoMRI, Hitachi Aloka Medical, Ltd., Tokyo, Japan).

### GLP-1 in vitro secretion assay

GLUTag cells were plated in 96-well plates in DMEM supplemented with 10% fetal bovine serum and antibiotics. For GLP-1 secretion, cells were washed twice with HBSS and incubated with oleic acid, monoolein, diolein or triolein at desired concentrations KRBH buffer containing 1 mM glucose. Two hour after incubation, the supernatants were collected and filtrated to remove the debris. GLP-1 content in the supernatant was determined by an ELISA method (WAKO, Osaka, Japan)

### Chronic dosing study in HFD- Streptozotocin (STZ) mice

Six-week-old male C57BL/6J mice were placed on 60% HFD. After 2 weeks of HFD feeding, the mice were intraperitoneally injected 90 mg/kg STZ. After 2 weeks, the mice were divided into vehicle or 30 mg/kg compA-treated groups so that each mean of body weight and plasma parameters had no statistically significant differences between the groups. Vehicle or compA was orally administrated once daily. BW and food intake were measured twice a week. Postprandial plasma parameters and blood GHb levels were measured every 2 weeks. Overnight fasting glucose and insulin levels were monitored after 5 weeks of compA administration. Homeostasis model assessment of insulin resistance (HOMA-IR) was calculated according to the following formula: [fasting plasma insulin level (μU/mL) × fasting plasma glucose (mg/dL)]/405 [21]. The liver, pancreas and small intestine were collected after 6-week dosing under 2% isoflurane anesthesia.

### Measurement of pancreatic insulin levels

Each isolated pancreas was weighed, homogenised in an ethanol/HCl solution and incubated overnight at 4°C. After removing the tissue debris by centrifugation, the supernatant was used to measure pancreatic insulin levels with a mouse insulin ELISA kit (Shibayagi, Gunma, Japan).

### Measurement of hepatic TG levels

Hepatic TG levels were determined by a colorimetric assay following lipid extraction with hexane/isopropanol. In brief, the tissue samples were homogenised in 6.7% sodium sulfate solution using a mixer mill (Retsch, Haan, Germany) and shaken vigorously after adding a hexane/isopropanol mixture (3:2). After centrifugation, the upper phase was transferred to a tube, evaporated under nitrogen gas and resuspended in isopropanol. Sample TG levels were analysed with the E-test Wako TG colorimetric assay kit (WAKO, Osaka, Japan).

### RNA extraction and quantitative PCR (qPCR) analysis

The small intestine was divided into two exact halves, and the weight of each part was measured. The intestinal mucosa was then collected for mRNA measurement. Total RNA was isolated using the RNeasy 96 Universal Tissue Kit (Qiagen Japan, Tokyo, Japan). RNA from each sample was converted to cDNA using the High Capacity RNA-to-cDNA Kit (Life Technologies Japan, Tokyo, Japan). Real-time quantitative PCR (SYBR green) was performed on a 7900HT Fast Real-Time PCR System (Life Technologies Japan, Tokyo, Japan). The primer sequences used are listed in S1 Table. Gene expression levels were normalised to Ppia.

### Statistical analysis

Statistical analysis was performed using SAS software (SAS Institute Inc., NC, USA). Data are expressed as mean + standard deviation (SD). Statistical analyses were performed using the one-tailed Williams’ test in dose-dependent studies; Two-tailed Student’s t-test, Aspin–Welch test or ANOVA followed by two-tailed Dunnett’s test or Steel’s test was used to analyse differences in single or multiple comparisons. *P*-values of <0.025 in the Williams’ test or <0.05 in all other tests were considered significant.

## Results

### Dose-dependent and long-lasting inhibitory effects of compA on postprandial hypertriglyceridemia

To confirm the in vivo efficacy of the MGAT2 inhibitor compA, changes in plasma TG levels were measured using MTT. CompA dose-dependently suppressed plasma TG elevation, and plasma CM/TG AUC in case of 10 mg/kg compA decreased by 50% compared with that observed in the case of the vehicle control (Fig 1B and 1C). CompA showed durable efficacy when 30 mg/kg was administered 16 h before the meal challenge (Fig 1D) and significantly reduced plasma CM/TG AUC by 58% (Fig 1E). Twenty-four hours after administration, compA plasma levels were 2.6 μmol/L, which were 1,000 times higher than the IC_50_ value measured with the mouse MGAT2 enzyme (S1 Fig). Fig 1D and the pharmacokinetics data suggested that daily dosing of 30 mg/kg compA could achieve 24 h of MGAT2 inhibition and modulate intestinal lipid metabolism.

### Alternative meal fat utilization due to MGAT2 inhibition

**Fig 2:**
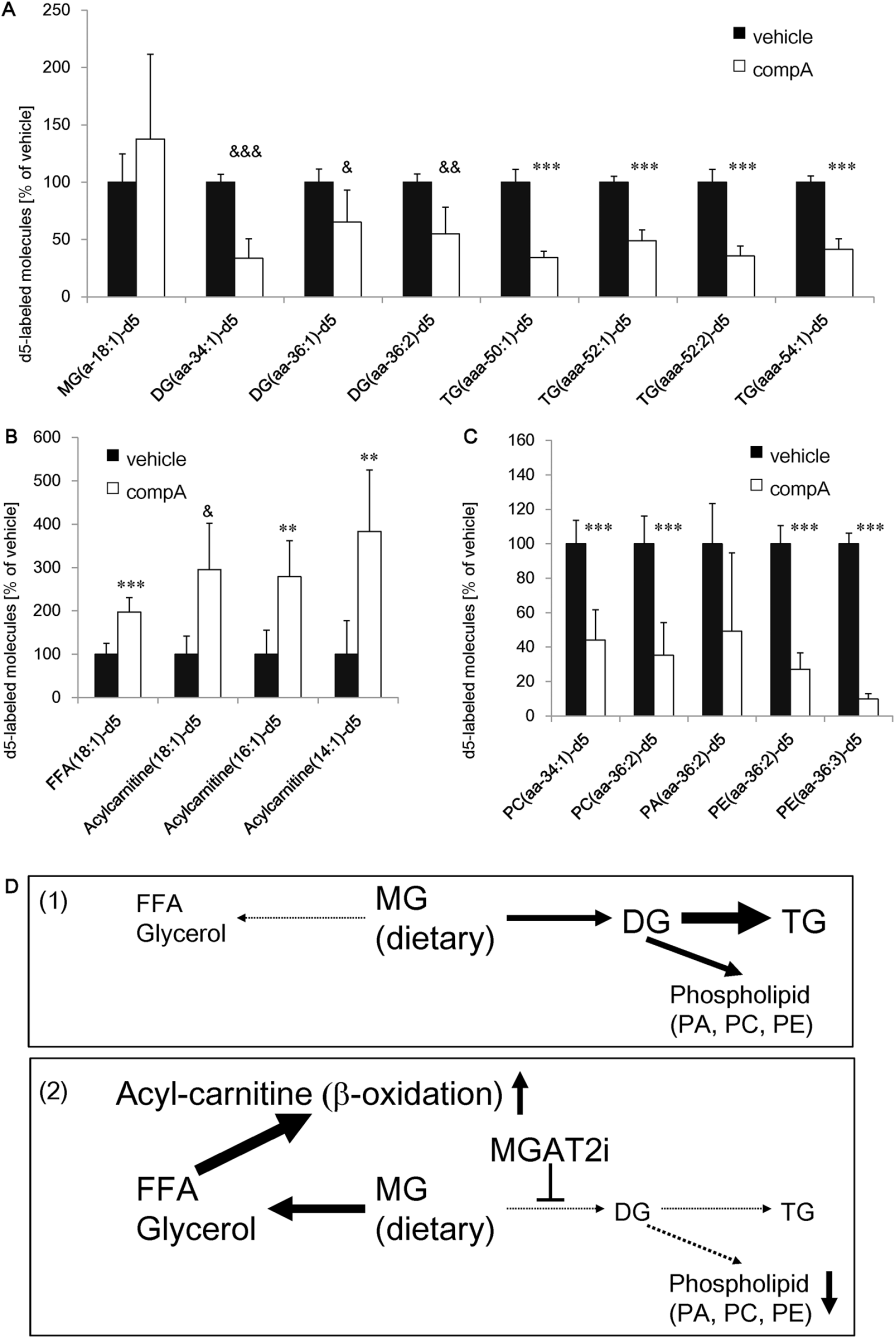
Lipid metabolic flux analysis of compA in intestinal mucosa. Fasted C57BL/6J mice were orally administered 30 mg/kg compA. Two hours after dosing, they were orally administered a liquid meal containing the ^2^H-labeled oleoylglycerol. Glycerol-labeled monoacylglycerol (MG) was used in panels 2A and 2C, and fatty acid-labeled MG was used in panel 2B as a tracer. (A) Intestinal ^2^H-labeled MG, diacylglycerol (DG), and triglyceride (TG) levels. (B) Intestinal ^2^H-labeled fatty acid and acylcarnitine levels. (C) Intestinal ^2^H-labeled phospholipids levels. n = 5. **: *P* < 0.01, ***: *P* < 0.001 vs. vehicle group by Student's t-test. &: *P* < 0.05, &&: *P* < 0.01, &&&: *P* < 0.001 vs. vehicle group by Aspin–Welch test. (D) Scheme of MGAT2 inhibitor-mediated intestinal fat metabolism. (1) Most of the dietary MG and free fatty acids (FFAs) is used as a source of TG resynthesis in enterocytes. Some of the synthesized DGs are pooled as phospholipids. (2) MGAT2 inhibitor suppresses DG/TG resynthesis. Transiently stored MG is hydrolyzed into glycerol and FFAs. The activated FFA (acylcarnitine) is transported into mitochondria as a substrate of β-oxidation.

To investigate changes in lipid metabolism with MGAT2 inhibition, compA-treated mice were subjected to intestinal lipid flux analysis with deuterium-labeled oleoylglycerol. CompA significantly decreased DGs (34:1, 36:1, 36:2) and TGs (50:1, 52:1, 52:2, 54:1) synthesized from labeled MG, together with a trend toward increased MG levels (Fig 2A). These data indicate that compA does not decrease MG absorption but inhibits MGAT2-dependent TG/DG resynthesis. In the lipid utilization analysis, compA significantly increased free fatty acid (FFA) and acylcarnitine levels. Increase in acylcarnitine (14:1) and (16:1) would be due to the enhancement of β-oxidation in small intestine of compA-treated mice (Fig 2B). CompA significantly decreased phosphatidic acid (PA), phosphatidylcholine (PC), and phosphoethanolamine (PE) levels (Fig 2C).

### Lipid-dependent effect of MGAT2 inhibition on short-term food intake

**Fig 3:**
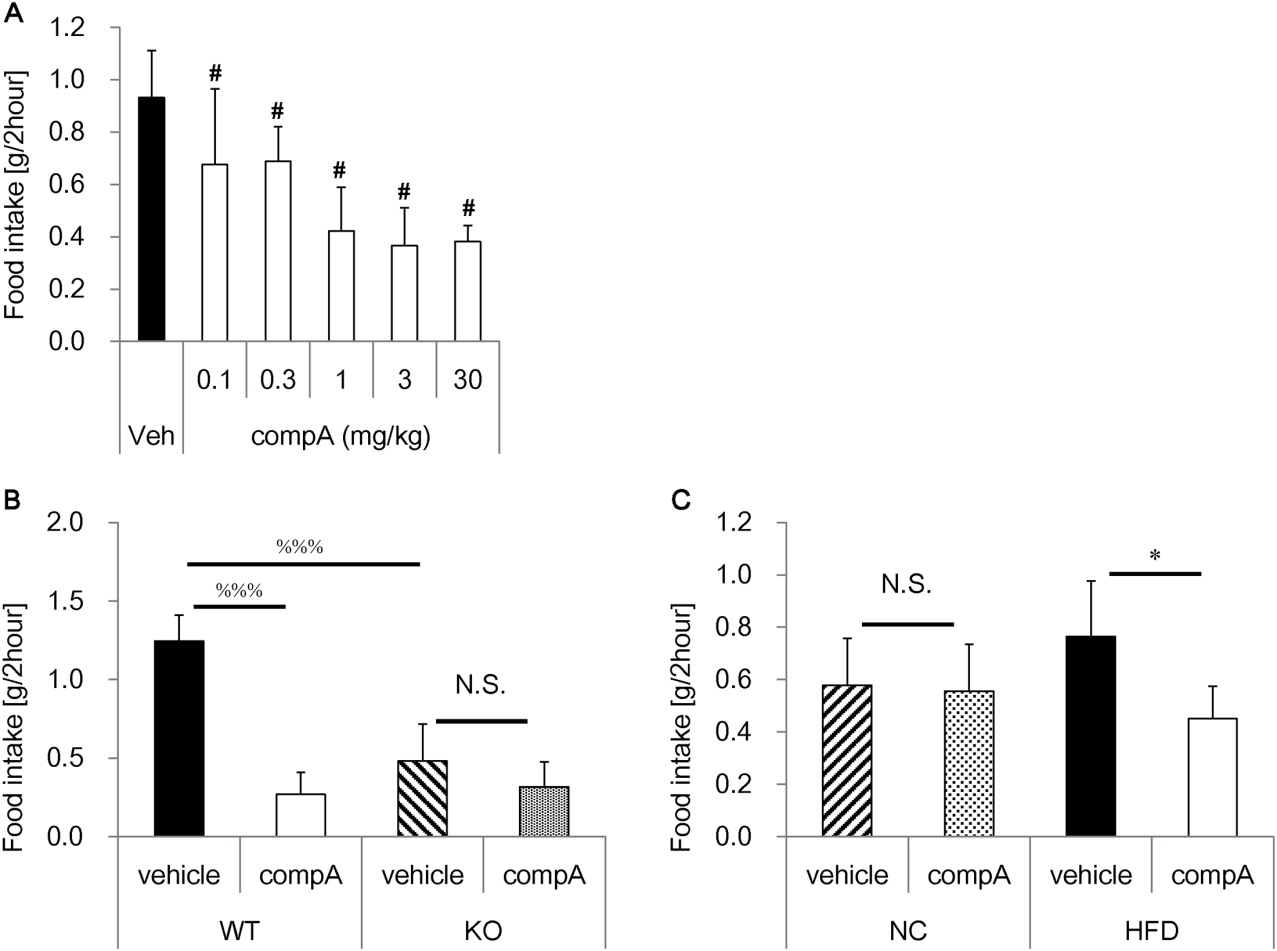
Acute anorectic effect of compA under high-fat diet (HFD)-feeding conditions. Fasted mice were orally administrated vehicle or compA and were fed either HFD or normal chow (NC) for 2 h. (A) Amount of HFD intake by C57BL/6J mice. (B) Amount of HFD intake by vehicle or 10 mg/kg compA-treated MGAT2 KO mice and WT littermates. (C) Amount of NC or HFD intake by vehicle or 10 mg/kg compA-treated C57BL/6J mice. n = 5 (A, B) or n = 7 (C). #: *P* < 0.025 vs. vehicle group by one-tailed Williams’ test. %%%: *P* < 0.001 vs. vehicle-administrated WT mice by Student’s t-test. *: *P* < 0.05 vs. HFD-fed vehicle group by Student's t-test. N.S.: not significant.

Anorectic effects of compA were investigated in C57BL/6J mice using a fasting–HFD refeeding regime. CompA significantly reduced food intake dose dependently, with 59% reduction at 30 mg/kg dosing (Fig 3A). Similar anorectic effects were observed in MGAT2 KO mice, and compA administration did not further decrease food intake (Fig 3B), indicating that compA-induced anorectic effects are MGAT2 dependent. Investigation of the relationship between food fat contents and anorectic effects revealed that the anorectic effects were specific to HFD, with no change in feeding under normal chow (NC) conditions (Fig 3C). This result indicates that compA does not cause conditioned taste aversion.

### Reduced body weight gain and adiposity in mice treated with compA

**Fig 4:**
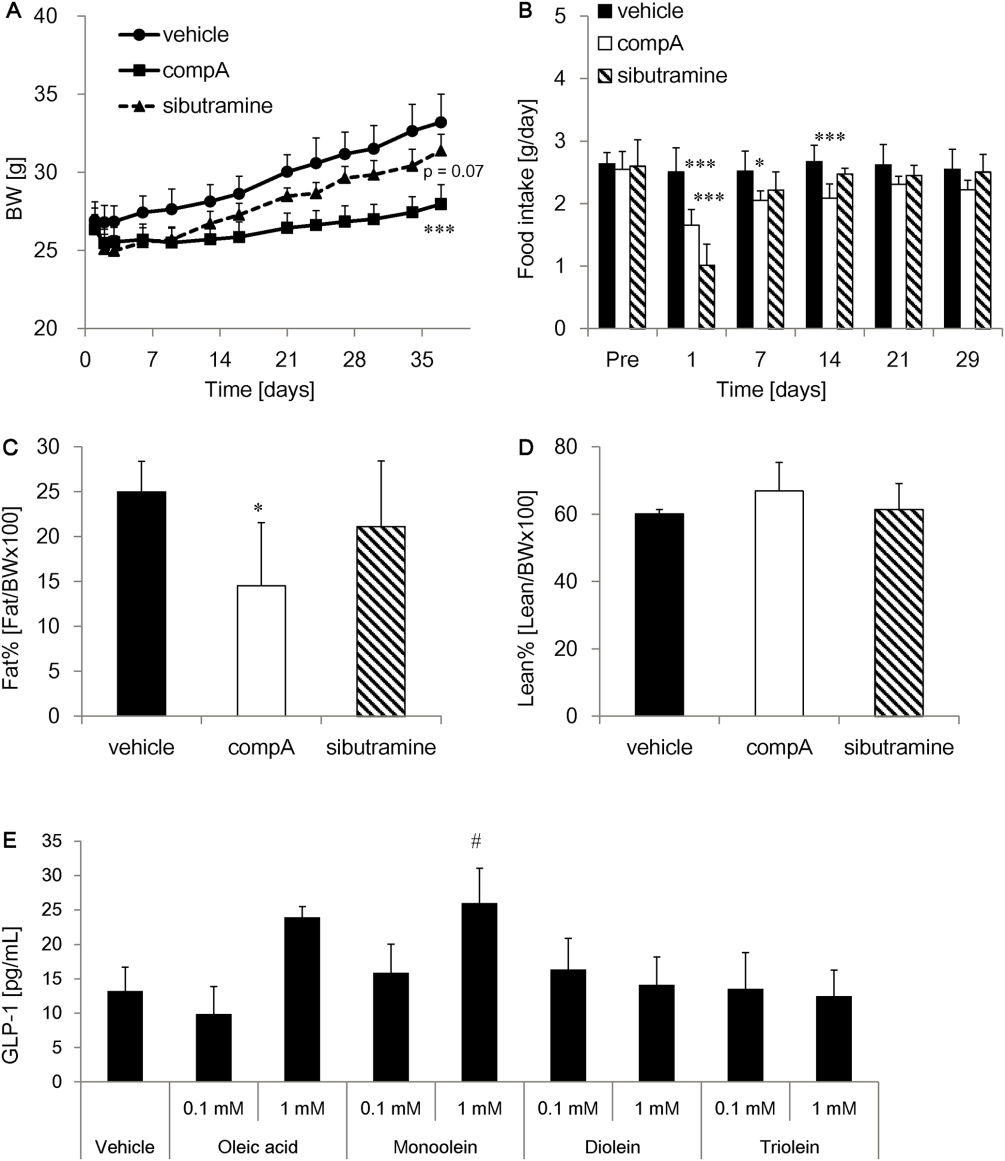
Anti-obesity effects of compA in C57BL/6J mice fed a high-fat diet (HFD). Male C57BL/6J mice were fed HFD and orally administered vehicle, 30 mg/kg compA, or 10 mg/kg sibutramine for 5 weeks. (A) Time course of changes in body weight (BW). (B) Food intake. Mean (C) fat mass and (D) lean mass. n = 6. *: *P* < 0.05, ***: *P* < 0.001 vs. WT mice by two-tailed Dunnett’s test. The results of Fig 4D were analyzed using two-tailed Steel’s test. (E) GLUTag cells were treated with assay buffer containing free fatty acid (oleic acid), monoacylglycerol (2-monoacylglycerol), diacylglycerol (diolein), and triglyceride (triolein) for 2h. Secreted GLP-1 levels in the assay buffer was measured by GLP-1 ELISA. n = 4. #: P < 0.025 vs. control by one-tailed Williams test.

Potential anti-obesity effects of compA were investigated in HFD-fed mice with repeated dosing for 37 days. At 30 mg/kg, compA significantly suppressed BW gains (Fig 4A). This effect was greater than that of sibutramine, which elicits anorectic effects through inhibition of serotonin and noradrenaline. However, the anorectic effect of compound A was less potent than that of sibutramine, especially early in the study (Fig 4B). Lean and fat mass were also measured using quantitative nuclear magnetic resonance analysis. CompA greatly suppressed fat mass increases with no significant impact on lean mass (Fig 4C and 4D), suggesting that BW suppression was predominantly due to fat mass reduction and not a growth disorder. In GLUTag enteroendocrine cells, MG but not DG nor TG increased GLP-1 secretion, suggesting MG increased by inhibition of MGAT2 could enhance the GLP-1 secretion (Fig 4E).

### Anti-diabetic effects of compA in HFD-STZ mice

**Fig 5:**
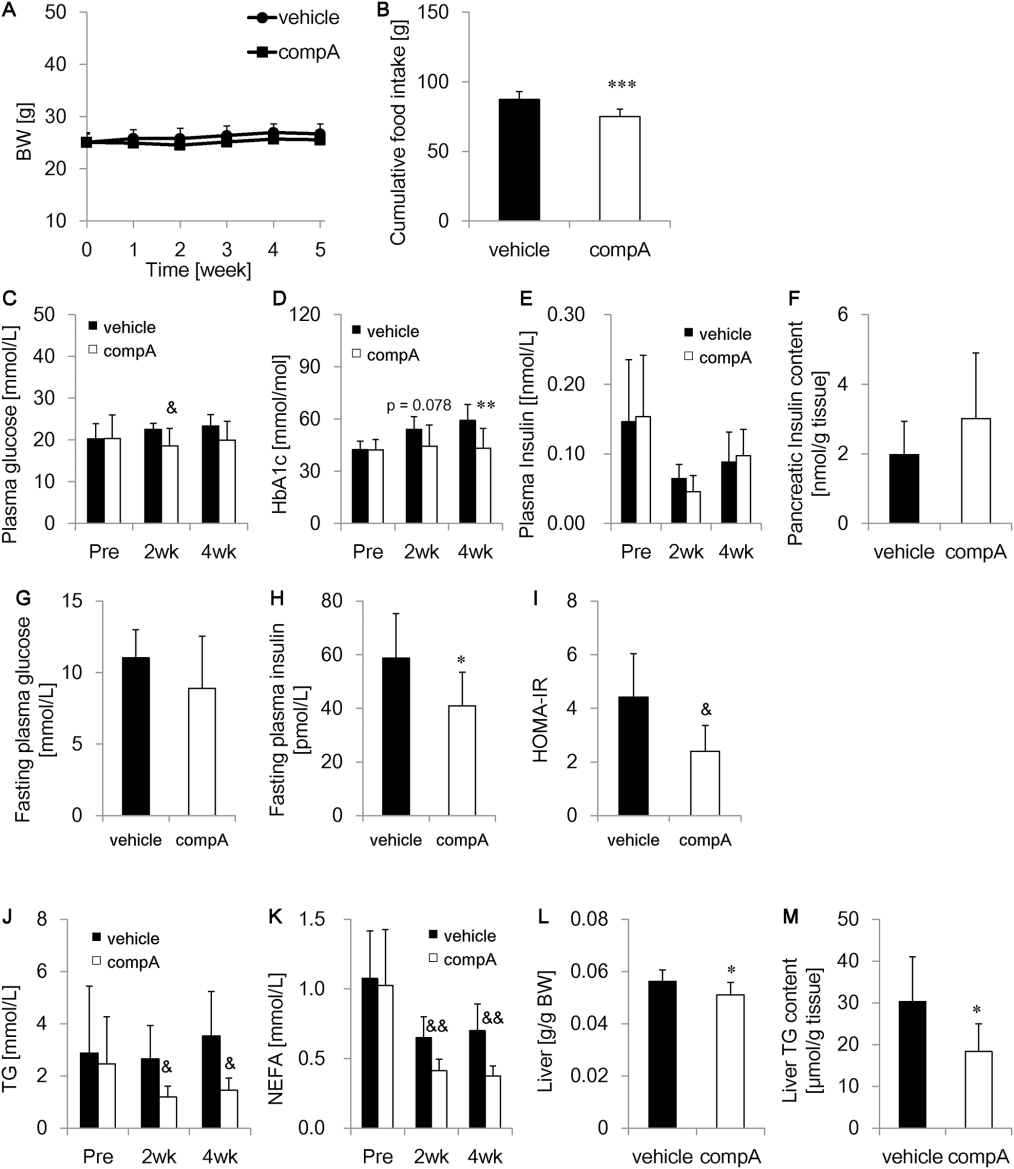
Anti-diabetic effect of compA in high-fat diet-streptozotocin (HFD-STZ) mice. Male HFD-STZ mice were orally administrated vehicle or 30 mg/kg compA for 6 weeks. Postprandial plasma parameters and blood GHb levels were measured every 2 weeks. Overnight fasting glucose and insulin levels were monitored after 5-week dosing. The liver, pancreas and small intestine were collected after 6-week dosing. (A) Time course of changes in body weight (BW). (B) Mean daily food intake. Mean (C) plasma glucose levels, (D) blood glycated hemoglobin (GHb) levels, and (E) plasma insulin levels before and 2 or 4 week after compA treatment. Mean (F) pancreatic insulin levels, (G) fasting plasma glucose levels, (H) plasma insulin levels, (I) HOMA-IR levels, (J) plasma triglyceride (TG) levels, (K) plasma non-esterified fatty acid (NEFA) levels, (L) liver weight, and (M) hepatic TG levels. n = 8. *: *P* < 0.05, **: *P* < 0.01, ***: *P* < 0.001 vs. the vehicle-treated group by Student’s t-test. &: *P* < 0.05, &&: *P* < 0.01 vs. the vehicle-treated group by Aspin–Welch test.

Anti-diabetic effects of compA were investigated in a 6-week treatment study in HFD-STZ mice. CompA did not significantly alter BW (Fig 5A) and only slightly decreased food intake (Fig 5B). Significant reductions in plasma glucose and blood GHb levels were observed in the compA-treated group (Fig 5C and 5D), whereas plasma insulin and pancreatic insulin levels were unchanged (Fig 5E and 5F). HOMA-IR was significantly decreased in compA-treated mice, with a trend toward decreasing fasting plasma glucose levels and significant reduction in plasma insulin levels (Fig 5G–5I). CompA significantly reduced plasma TG and NEFA levels (Fig 5J and 5K) and liver weight and hepatic TG levels (Fig 5L and 5M). Thus, these results suggest that compA improves plasma glycemic and lipid metabolism as well as hepatic steatosis, with amelioration of insulin resistance in HFD-STZ mice.

### Reprogramming of intestinal structure and gene expressions by compA treatment

**Fig 6:**
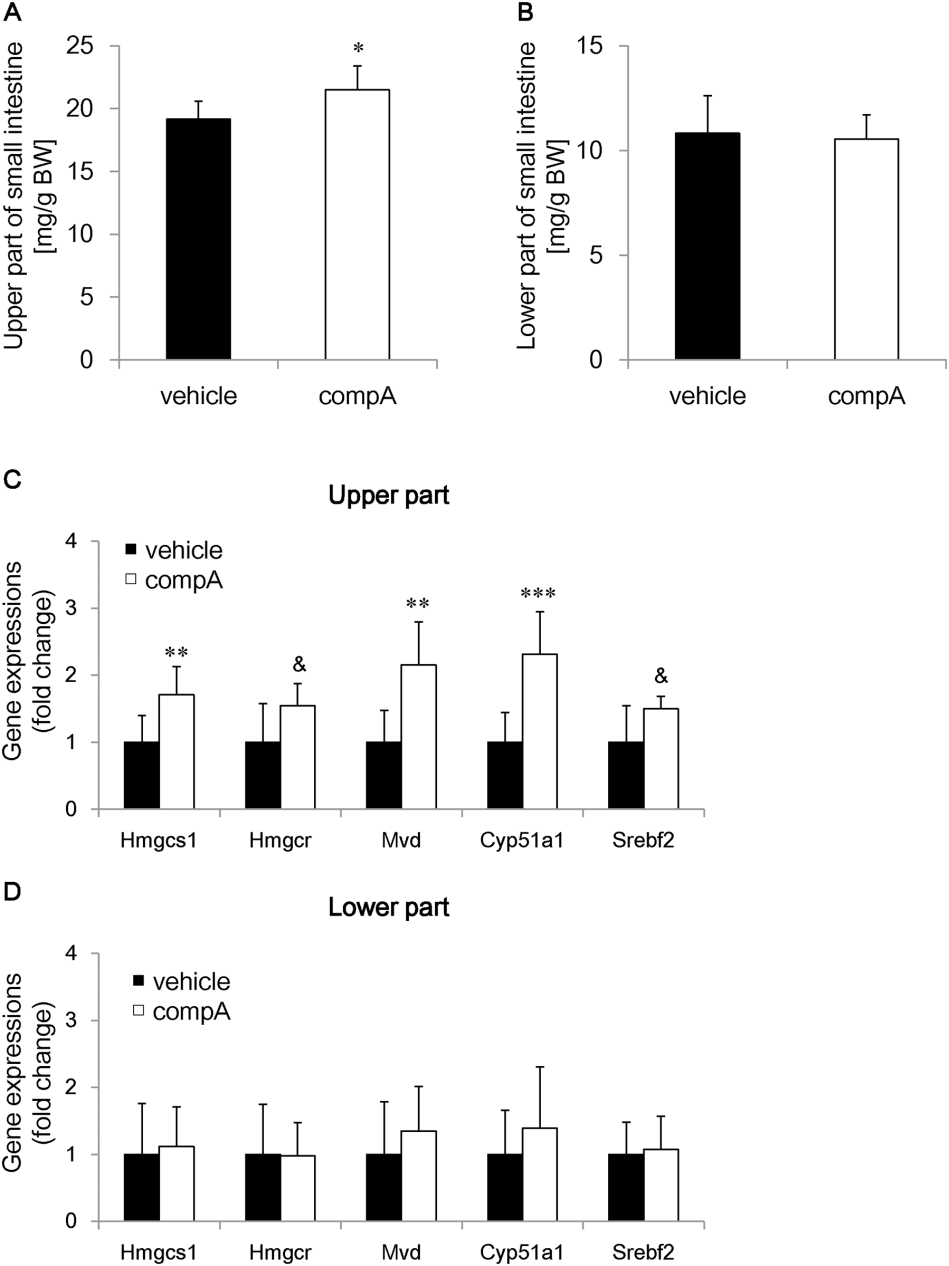
Intestinal remodeling by compA in high-fat diet-streptozotocin (HFD-STZ) mice. The small intestine of HFD-STZ mice was collected after 6-week administration of 30 mg/kg compA. Mean (A) tissue weight of the upper and (B) lower halves of the small intestine. Mean levels of gene expression for select enzymes involved in cholesterol metabolism in the (C) upper and (D) lower halves of the small intestine. n = 7 (for the weight of the upper intestine) or n = 8. *: *P* < 0.05, **: *P* < 0.01, ***: *P* < 0.001 vs. the vehicle-treated group by Student’s t-test. &: *P* < 0.05 vs. the vehicle-treated group by Aspin–Welch test.

Effects on the small intestine weight and gene expression were investigated in compA-treated HFD-STZ mice. CompA significantly increased the intestinal weight of the upper half without altering the lower half (Fig 6A and 6B). CompA-treated mice also showed increases in gene expression for enzymes involved in cholesterol synthesis (Hmgcs1, Hmgcr, Mvd, Cyp51a1, and Srebf2), which were reported to facilitate structural remodeling of the small intestine [22], in the upper part of the small intestine (Fig 6C) but not in the lower part (Fig 6D).

## Discussion

In this report, we describe for the first time the pharmacological effects of compA, a selective MGAT2 inhibitor. CompA inhibited 17% of HFD-induced body weight gain during 5 weeks of treatment. In previous studies, MGAT2 KO mice showed body weight reduction by approximately 2.5% per week compared with WT littermates under HFD-feeding conditions [18, 19]. Our data demonstrated comparable anti-obesity effects of compA relative to MGAT2 genetic deletion. Fig 2B indicated that compA increases β-oxidation in the small intestine. Yen's report indicates that MGAT2 inhibition increased postprandial energy expenditure [18]. These results suggest that compA exhibits anti-obesity effects through increasing energy expenditure by altering intestinal postprandial lipid utilization.

Along with an increase in energy expenditure by compA, our study revealed that MGAT2 inhibition by gene ablation or the small molecule compound moderately reduced food intake under HFD-feeding conditions. Our data and other reports indicate that MGAT2 inhibitor-induced anorectic effects occur only under HFD-feeding conditions [23, 24]. Therefore, an MGAT2 inhibitor could ameliorate unbalanced eating habits with fat palatability and reduction of high-fat food intake could contribute to the anti-obesity effects of the MGAT2 inhibitor. We postulate that inhibition of intestinal MGAT2 activity regulates systemic energy intake via hormonal and neural signals in response to dietary fat and fat metabolites in the intestinal lumen. In previous reports, the spatial distribution of fat absorption in the intestine was altered in MGAT2 KO mice, with increased plasma anorectic hormones, including GLP-1 and PYY levels [18, 19]. As shown in Fig 4E, MG but not DG nor TG increased GLP-1 secretion in GLUTag enteroendocrine cells, suggesting that MGAT2 inhibitor could enhance GLP-1 secretion through the increase of intestinal 2-monoacylglycerol levels. Together with these data and reports, it is likely that the increase in these gut peptides via MGAT2 inhibition contributes to food intake reduction. Moreover, MGAT2 exhibits high preference toward MGs with long-chain unsaturated fatty acids [13]. One specific MG, sn-2-arachidonoylglycerol, has been identified as an endogenous ligand for cannabinoid receptors [25, 26]. Fig 2A suggests that compA could increase intestinal MG levels. Thus, it is possible that MGAT2 regulates food intake by modulating central endocannabinoid signaling. Although it is presumed that the substrate preference would mediate the amount of 2-arachidonoyl-MG and endocannabinoid signaling [27], our study has not measured the intracerebral concentration of 2-arachidonoylglycerol and compA. Further studies are required to identify the precise underlying mechanisms.

Several studies have described the intestinal and extraintestinal functions of MGAT2. Yen et al. recently demonstrated that intestine-specific MGAT2 KO mice showed a decrease in fat absorption, similar to that observed with systemic ablation of MGAT2 genes [23]. They showed somewhat weaker anti-obesity phenotypes, including increased energy expenditure and protection against HFD-induced body weight gain, than that observed in systemic KO mice. In contrast, amelioration of obesity-associated comorbidities, including hepatic steatosis and insulin resistance, was nearly the same as that in systemic KO mice. Concurrently, intestine-specific rescue of MGAT2 expression in MGAT2 KO mice almost completely reversed the amelioration of hepatic steatosis [28]. Mice with intestine-specific MGAT2 expression showed higher energy expenditure than WT mice and partially suppressed HFD-induced BW gain, indicating that MGAT2 activities in extraintestinal tissues may also modulate systemic energy balance. CompA showed improvement in plasma glucose and lipid parameters and a decrease in hepatic TG levels with durable systemic exposure after oral administration, suggesting that compA could not only mediate intestinal lipid absorption but also ameliorate extraintestinal fat utilization and insulin sensitivity.

Furthermore, our study demonstrated that genetic ablation of MGAT2 (S2 Fig) and compA improved HFD-STZ-induced hyperglycemia and insulin resistance. Although MGAT2 inhibition by compA had little effect on plasma and pancreatic insulin levels, it clearly decreased HOMA-IR and improved peripheral insulin sensitivity. CompA treatment significantly reduced plasma TG and NEFA levels and hepatic TG levels in HFD-STZ mice. Amelioration of postprandial dyslipidemia by MGAT2 inhibition is one possible mechanism for improving insulin sensitivity via attenuation of ectopic lipotoxicity in peripheral tissues. Fig 3 indicated that MGAT2 inhibition decreased the food intake when first exposed to HFD. On the other hand, Fig 4B and S2 Fig suggest that MGAT2-mediated reduction of food intake is abolished soon after chronic HFD feeding. These data coincide with the recent reports [23, 24]. Further study are needed in order to evaluate whether the transient change in food intake effect the glucose metabolism after chronic administration.

Another hypothesis is that MGAT2 inhibition generates a new site for expending excessive energy and glucose. CompA-treated mice showed the increase in tissue weight and gene expression involved in cholesterol synthesis in the upper small intestine. Intestinal remodeling is observed in the Roux limb of Roux-en-Y gastric bypass (RYGB)-treated animals and postoperatively in humans [22, 29]. RYGB leads to substantial and sustained systemic metabolic improvement and weight loss. As is well known, glycemic improvement with RYGB surgery is beyond that anticipated by weight loss alone [30]. The increase in carbohydrate/fat utilization via intestinal remodeling is a more convincing hypothesis that may explain the hypoglycemic effect of RYGB [22]. In a previous study, MGAT2 KO mice absorbed less fat in the proximal small intestine [18]. The intestinal hyperplasia observed with compA treatment could occur in response to a decrease in luminal fat absorption from the proximal gut and could potentially increase nutrient absorption through the basolateral side. Measurement of intestinal glucose uptake would be needed to determine the effect of the intestinal remodeling by MGAT2 inhibition [22].

Pancreatic lipase inhibitor and DGAT1 inhibitor have been investigated as agents modifying intestinal fat absorption. Orlistat, which prevents fat absorption via pancreatic lipase inhibition, is marketed as an anti-obesity agent. However, use of orlistat is limited due to steatorrhea and gastrointestinal (GI) side effects [31]. We observed that compA did not increase fecal fat excretion (S3 Fig), suggesting that MGAT2 inhibitors should avoid these adverse effects in preclinical studies and clinical trials. DGAT1 inhibitors, which prevent downstream MGAT2-mediated TG resynthesis in the small intestine, have been preclinically and clinically investigated [32, 33]. Several clinical trials revealed that DGAT1 inhibitors prevented postprandial TG elevation via inhibition of intestinal DGAT activity [33, 34]. However, subjects who were administered the DGAT1 inhibitor experienced several GI adverse events, including vomiting, abdominal pain, and diarrhea [32–34]. These results are consistent with a recent clinical case report in which children with congenital DGAT1 deficiency presented severe, intractable diarrhea soon after birth [35]. Liu et al. demonstrated that a DGAT1 inhibitor increased intracellular PC levels both in vitro and in vivo [36]. Several reports have stated that PC can cause GI disorders, including diarrhea, loose stools and nausea [37–39]. These actions of PC would cause the GI adverse effects observed in subjects on DGAT1 inhibitors. In contrast, our study found that compA, which exhibits IC_50_ values of >10 μmol/L against DGAT1 [20], does not increase intestinal PC levels due to the inhibition of DG, precursor of phospholipids, synthesis (Fig 2D). This suggests that MGAT2 could avoid PC-stimulated GI disorders, although additional studies are required to clarify the relationships between the metabolite related to MGAT2 inhibition and GI disorders. In clinical trials, DGAT1 inhibitors have not shown precise anti-obesity and anti-diabetic effects, because the drug doses are limited, at least in part, due to lack of an adequate therapeutic window between the efficacy dose and the dose causing the GI adverse effects [32–34]. We anticipate that a MGAT2 inhibitor would ameliorate obesity and diabetes without PC-stimulated GI toxicity in clinical trials.

Another possible indication of MGAT2 inhibitors is treatment of dyslipidemia caused by genetic disorders. Familial chylomicronemia syndrome (FCS) with extreme hypertriglyceridemia is a disorder of lipoprotein metabolism due to loss-of-function mutations in the gene encoding LPL or ApoC-II [40]. We conducted MTT with administration of an LPL inhibitor, which simulates the pathology of FCS. CompA prevented postprandial TG elevation under LPL-deficient conditions (Fig 1). FCS patients usually suffer from severe abdominal pain caused by acute pancreatitis. FCS also manifests as eruptive xanthomas, hepatosplenomegaly, foam cell infiltration of bone marrow, and lipemia retinalis. Presently, there is no cure for FCS except for genetic modification by adeno-associated virus [41, 42]. CompA is expected to provide a therapeutic option for the treatment of this severe untreated disease.

In conclusion, the present study demonstrates that the selective and potent MGAT2 inhibitor compA remarkably ameliorates dyslipidemia, obesity, and diabetes. These effects are based on totally different mechanisms from existing drugs, involving changes in intestinal lipid absorption and utilization. CompA administration induced intestinal remodeling as observed in patients with bariatric surgeries, which are among the most effective treatments for diabetes and obesity at present. Our findings revealed the relationship between alteration of fat utilization and bariatric surgery and showed MGAT2 inhibition as a promising molecular target with non-invasive bariatric surgery-mimetic effects.

## Supporting Information

S1 Fig Pharmacokinetics of compA in mice. Fifty-four-week-old male C57BL/6J mice fed 45% HFD were orally administered 30 mg/kg compA suspension. Blood samples were collected at 0.25, 0.5, 1, 2, 4, 8, and 24 h after single administration. The plasma was isolated by centrifugation, and the samples were mixed with acetonitrile and centrifuged. The supernatants were diluted with an appropriate volume of 0.2% (v/v) formic acid in 10 mmol/L ammonium formate. Plasma compA levels were measured using liquid chromatography with tandem mass spectrometry (LC/MS/MS). LC/MS/MS was conducted with an API 5000 triple quadruple mass spectrometer (Applied Biosystems, Foster City, CA) coupled with a turbo ion spray interface in the positive ion mode and connected with UFLC (Shimadzu, Kyoto, Japan). Reverse-phase chromatography [mobile phase A, 0.2% (v/v) formic acid in 10 mmol/L ammonium formate; mobile phase B, acetonitrile] was used to elute and separate the different substrates with a Shim-pack XR-ODS C18 column (20 mm × 2.0 mm, 5 μm, Shimazu Co., Ltd.). Plasma concentrations at 0.25, 0.5, 1, 2, 4, 8, and 24 h after oral administration of compA at a dose of 30 mg/kg. n = 3.
(TIF)

S2 Fig Effect of MGAT2 gene ablation on high-fat-diet (HFD)-streptozotocin (STZ)-induced hyperglycemia. Twenty-two-week-old male MGAT2 KO mice and WT littermates were placed on 60% HFD. After 2 weeks of HFD feeding, the mice were intraperitoneally injected 90 mg/kg STZ (Sigma-Aldrich Japan, Tokyo, Japan). BW, plasma parameters, and blood GHb levels were monitored every 2 weeks. Food intake was measured 7 days after STZ administration. The pancreas was collected 6 weeks after STZ dosing under 2% isoflurane anesthesia. (A) BW. (B) Mean food intake 7 days after STZ administration. (C) Time course of changes in plasma glucose levels, (D) blood glycated hemoglobin (GHb) levels and (E) plasma insulin levels. (F) Mean pancreatic insulin levels. n = 10 (WT) or n = 11 (KO). *: *P*< 0.05, **: *P*< 0.01, ***: *P*< 0.001 vs. WT mice by Student’s t-test. &: *P*< 0.05, &&&: *P*< 0.001 vs. WT mice by Aspin–Welch test.
(TIF)

S3 Fig Effect of compA on fecal lipid levels. Fecal fat levels were determined by a colorimetric assay following lipid extraction with hexane/isopropanol. In brief, the fecal samples were homogenised in hexane/isopropanol mixture (3:2) using a mixer mill (Retsch, Haan, Germany) and shaken vigorously. After centrifugation, the supernatant was transferred to a tube, evaporated under nitrogen gas and resuspended in isopropanol. Sample triglyceride, cholesterol and fatty acid levels were analysed with the E-test Wako colorimetric assay kits (WAKO, Osaka, Japan). (A) Fecal triglyceride levels. (B) Fecal cholesterol levels. (C) Fecal fatty acid levels. n = 6.
(TIF)

S1 Table Primer sequences for quantitative PCR. (XLSX)

S2 Table Data file represents the values and standard deviations of each figures. (XLSX)

## References

[1] Friedman JM. Obesity in the new millennium. Nature. 2000;404(6778):632–4. 10.1038/35007504 . 10766249

[2] Spiegelman BM, Flier JS. Adipogenesis and obesity: rounding out the big picture. Cell. 1996;87(3):377–89. . 8898192 10.1016/s0092-8674(00)81359-8

[3] Bistrian BR. Biochemical and Physiological Aspects of Human Nutrition. The American Journal of Clinical Nutrition. 2000;71(6):1619–20.

[4] McQuaid SE, Hodson L, Neville MJ, Dennis AL, Cheeseman J, Humphreys SM, et al Downregulation of adipose tissue fatty acid trafficking in obesity: a driver for ectopic fat deposition? Diabetes. 2011;60(1):47–55. 10.2337/db10-0867 20943748 PMC3012196

[5] Byrne CD, Targher G. Ectopic fat, insulin resistance, and nonalcoholic fatty liver disease: implications for cardiovascular disease. Arteriosclerosis, thrombosis, and vascular biology. 2014;34(6):1155–61. 10.1161/ATVBAHA.114.303034 . 24743428

[6] Lara-Castro C, Garvey WT. Intracellular lipid accumulation in liver and muscle and the insulin resistance syndrome. Endocrinology and metabolism clinics of North America. 2008;37(4):841–56. 10.1016/j.ecl.2008.09.002 19026935 PMC2621269

[7] Takeuchi K, Reue K. Biochemistry, physiology, and genetics of GPAT, AGPAT, and lipin enzymes in triglyceride synthesis. American journal of physiology Endocrinology and metabolism. 2009;296(6):E1195–209. 10.1152/ajpendo.90958.2008 19336658 PMC2692402

[8] Coleman RA, Mashek DG. Mammalian triacylglycerol metabolism: synthesis, lipolysis, and signaling. Chemical reviews. 2011;111(10):6359–86. 10.1021/cr100404w 21627334 PMC3181269

[9] Yamashita A, Hayashi Y, Nemoto-Sasaki Y, Ito M, Oka S, Tanikawa T, et al Acyltransferases and transacylases that determine the fatty acid composition of glycerolipids and the metabolism of bioactive lipid mediators in mammalian cells and model organisms. Progress in lipid research. 2014;53:18–81. 10.1016/j.plipres.2013.10.001 24125941

[10] Coleman RA, Haynes EB. Monoacylglycerol acyltransferase. Evidence that the activities from rat intestine and suckling liver are tissue-specific isoenzymes. The Journal of biological chemistry. 1986;261(1):224–8. . 3001050

[11] Bell RM, Coleman RA. Enzymes of glycerolipid synthesis in eukaryotes. Annual review of biochemistry. 1980;49:459–87. 10.1146/annurev.bi.49.070180.002331 . 6250446

[12] Yen CL, Stone SJ, Cases S, Zhou P, Farese RV Jr. Identification of a gene encoding MGAT1, a monoacylglycerol acyltransferase. Proceedings of the National Academy of Sciences of the United States of America. 2002;99(13):8512–7. 10.1073/pnas.132274899 12077311 PMC124292

[13] Yen CL, Farese RV Jr., MGAT2, a monoacylglycerol acyltransferase expressed in the small intestine. The Journal of biological chemistry. 2003;278(20):18532–7. 10.1074/jbc.M301633200 . 12621063

[14] Cao J, Lockwood J, Burn P, Shi Y. Cloning and functional characterization of a mouse intestinal acyl-CoA:monoacylglycerol acyltransferase, MGAT2. The Journal of biological chemistry. 2003;278(16):13860–6. 10.1074/jbc.M300139200 . 12576479

[15] Cheng D, Nelson TC, Chen J, Walker SG, Wardwell-Swanson J, Meegalla R, et al Identification of acyl coenzyme A:monoacylglycerol acyltransferase 3, an intestinal specific enzyme implicated in dietary fat absorption. The Journal of biological chemistry. 2003;278(16):13611–4. 10.1074/jbc.C300042200 . 12618427

[16] Yue YG, Chen YQ, Zhang Y, Wang H, Qian YW, Arnold JS, et al The acyl coenzymeA:monoacylglycerol acyltransferase 3 (MGAT3) gene is a pseudogene in mice but encodes a functional enzyme in rats. Lipids. 2011;46(6):513–20. 10.1007/s11745-011-3537-1 . 21312067

[17] Senior JR, Isselbacher KJ. Direct esterification of monoglycerides with palmityl coenzyme A by intestinal epithelial subcellular fractions. The Journal of biological chemistry. 1962;237:1454–9. . 13910673

[18] Yen CL, Cheong ML, Grueter C, Zhou P, Moriwaki J, Wong JS, et al Deficiency of the intestinal enzyme acyl CoA:monoacylglycerol acyltransferase-2 protects mice from metabolic disorders induced by high-fat feeding. Nature medicine. 2009;15(4):442–6. 10.1038/nm.1937 19287392 PMC2786494

[19] Tsuchida T, Fukuda S, Aoyama H, Taniuchi N, Ishihara T, Ohashi N, et al MGAT2 deficiency ameliorates high-fat diet-induced obesity and insulin resistance by inhibiting intestinal fat absorption in mice. Lipids in health and disease. 2012;11:75 10.1186/1476-511X-11-75 22698140 PMC3474177

[20] Sato K, Takahagi H, Kubo O, Hidaka K, Yoshikawa T, Kamaura M, et al Optimization of a novel series of N-phenylindoline-5-sulfonamide-based acyl CoA:monoacylglycerol acyltransferase-2 inhibitors: Mitigation of CYP3A4 time-dependent inhibition and phototoxic liabilities. Bioorg Med Chem. 2015 10.1016/j.bmc.2015.06.003 . 26100443

[21] Matthews DR, Hosker JP, Rudenski AS, Naylor BA, Treacher DF, Turner RC. Homeostasis model assessment: insulin resistance and beta-cell function from fasting plasma glucose and insulin concentrations in man. Diabetologia. 1985;28(7):412–9. . 3899825 10.1007/BF00280883

[22] Saeidi N, Meoli L, Nestoridi E, Gupta NK, Kvas S, Kucharczyk J, et al Reprogramming of intestinal glucose metabolism and glycemic control in rats after gastric bypass. Science. 2013;341(6144):406–10. 10.1126/science.1235103 23888041 PMC4068965

[23] Nelson DW, Gao Y, Yen MI, Yen CL. Intestine-specific deletion of acyl-CoA:monoacylglycerol acyltransferase (MGAT) 2 protects mice from diet-induced obesity and glucose intolerance. The Journal of biological chemistry. 2014;289(25):17338–49. 10.1074/jbc.M114.555961 24784138 PMC4067168

[24] Banh T, Nelson DW, Gao Y, Huang T-N, Yen M-I, Yen C-LE. Adult-onset deficiency of acyl CoA: monoacylglycerol acyltransferase 2 protects mice from diet-induced obesity and glucose intolerance. Journal of lipid research. 2015;56(2):379–89. 10.1194/jlr.M055228 25535286 PMC4306691

[25] Stella N, Schweitzer P, Piomelli D. A second endogenous cannabinoid that modulates long-term potentiation. Nature. 1997;388(6644):773–8. 9285589 10.1038/42015

[26] Sugiura T, Kodaka T, Nakane S, Miyashita T, Kondo S, Suhara Y, et al Evidence That the Cannabinoid CB1 Receptor Is a 2-Arachidonoylglycerol Receptor STRUCTURE-ACTIVITY RELATIONSHIP OF 2-ARACHIDONOYLGLYCEROL, ETHER-LINKED ANALOGUES, AND RELATED COMPOUNDS. Journal of Biological Chemistry. 1999;274(5):2794–801. 9915812 10.1074/jbc.274.5.2794

[27] Yen CL, Nelson DW, Yen MI. Intestinal triacylglycerol synthesis in fat absorption and systemic energy metabolism. Journal of lipid research. 2015;56(3):489–501. 10.1194/jlr.R052902 25231105 PMC4340298

[28] Gao Y, Nelson DW, Banh T, Yen MI, Yen CL. Intestine-specific expression of MOGAT2 partially restores metabolic efficiency in Mogat2-deficient mice. Journal of lipid research. 2013;54(6):1644–52. 10.1194/jlr.M035493 23536640 PMC3646465

[29] Spak E, Bjorklund P, Helander HF, Vieth M, Olbers T, Casselbrant A, et al Changes in the mucosa of the Roux-limb after gastric bypass surgery. Histopathology. 2010;57(5):680–8. 10.1111/j.1365-2559.2010.03677.x . 21054493

[30] Goldfine AB, Patti ME. Diabetes improvement following Roux-en-Y gastric bypass: understanding dynamic changes in insulin secretion and action. Diabetes. 2014;63(5):1454–6. 10.2337/db13-1918 24757197 PMC3994963

[31] Yanovski SZ, Yanovski JA. Long-term drug treatment for obesity: a systematic and clinical review. Jama. 2014;311(1):74–86. 10.1001/jama.2013.281361 24231879 PMC3928674

[32] Novartis. A 12-week multi-center, randomized, double-blind, placebocontrolled, parallel-group adaptive design study to evaluate the efficacy on blood glucose control and safety of five doses of LCQ908 (2, 5, 10, 15 and 20mg) or sitagliptin 100 mg on a background therapy of metformin in obese patients with type 2 diabetes. 2010. Available: http://www.novctrd.com/ctrdWebApp/clinicaltrialrepository/displayFile.do?trialResult=4313.

[33] Denison H, Nilsson C, Kujacic M, Lofgren L, Karlsson C, Knutsson M, et al Proof of mechanism for the DGAT1 inhibitor AZD7687: results from a first-time-in-human single-dose study. Diabetes, obesity & metabolism. 2013;15(2):136–43. 10.1111/dom.12002 . 22950654

[34] Denison H, Nilsson C, Lofgren L, Himmelmann A, Martensson G, Knutsson M, et al Diacylglycerol acyltransferase 1 inhibition with AZD7687 alters lipid handling and hormone secretion in the gut with intolerable side effects: a randomized clinical trial. Diabetes, obesity & metabolism. 2014;16(4):334–43. 10.1111/dom.12221 . 24118885

[35] Haas JT, Winter HS, Lim E, Kirby A, Blumenstiel B, DeFelice M, et al DGAT1 mutation is linked to a congenital diarrheal disorder. The Journal of clinical investigation. 2012;122(12):4680 10.1172/JCI64873 23114594 PMC3533555

[36] Liu J, Gorski JN, Gold SJ, Chen D, Chen S, Forrest G, et al Pharmacological inhibition of diacylglycerol acyltransferase 1 reduces body weight and modulates gut peptide release—Potential insight into mechanism of action. Obesity. 2013;21(7):1406–15. 10.1002/oby.20193 23671037

[37] Kidd P. Phosphatidylcholine (monograph). Alternative Medicine Review Monographs Volume One Dover, ID: Thorne Research, Inc. 2002:310–5.

[38] Calorini L, Mugnai G, Mannini A, Ruggieri S. Effect of phosphatidylcholine structure on the adenylate cyclase activity of a murine fibroblast cell line. Lipids. 1993;28(8):727–30. . 8377588 10.1007/BF02535994

[39] Domino EF, May WW, Demetriou S, Mathews B, Tait S, Kovacic B. Lack of clinically significant improvement of patients with tardive dyskinesia following phosphatidylcholine therapy. Biological psychiatry. 1985;20(11):1189–96. . 2864962 10.1016/0006-3223(85)90177-5

[40] Jameson JL. Harrison's endocrinology: McGraw-Hill Medical; 2010.

[41] Carpentier AC, Frisch F, Labbe SM, Gagnon R, de Wal J, Greentree S, et al Effect of alipogene tiparvovec (AAV1-LPL(S447X)) on postprandial chylomicron metabolism in lipoprotein lipase-deficient patients. The Journal of clinical endocrinology and metabolism. 2012;97(5):1635–44. 10.1210/jc.2011-3002 . 22438229

[42] Scott LJ. Alipogene Tiparvovec: A Review of Its Use in Adults with Familial Lipoprotein Lipase Deficiency. Drugs. 2015:1–8. 25559420 10.1007/s40265-014-0339-9

