# Supplementary figures and images for: Pharmacological Inhibition of Monoacylglycerol O-Acyltransferase 2 Improves Hyperlipidemia, Obesity, and Diabetes by Change in Intestinal Fat Utilization
Source: PLoS One. 2016 Mar 3;11(3):e0150976. doi: 10.1371/journal.pone.0150976 (PMC4777574; doi:10.1371/journal.pone.0150976)

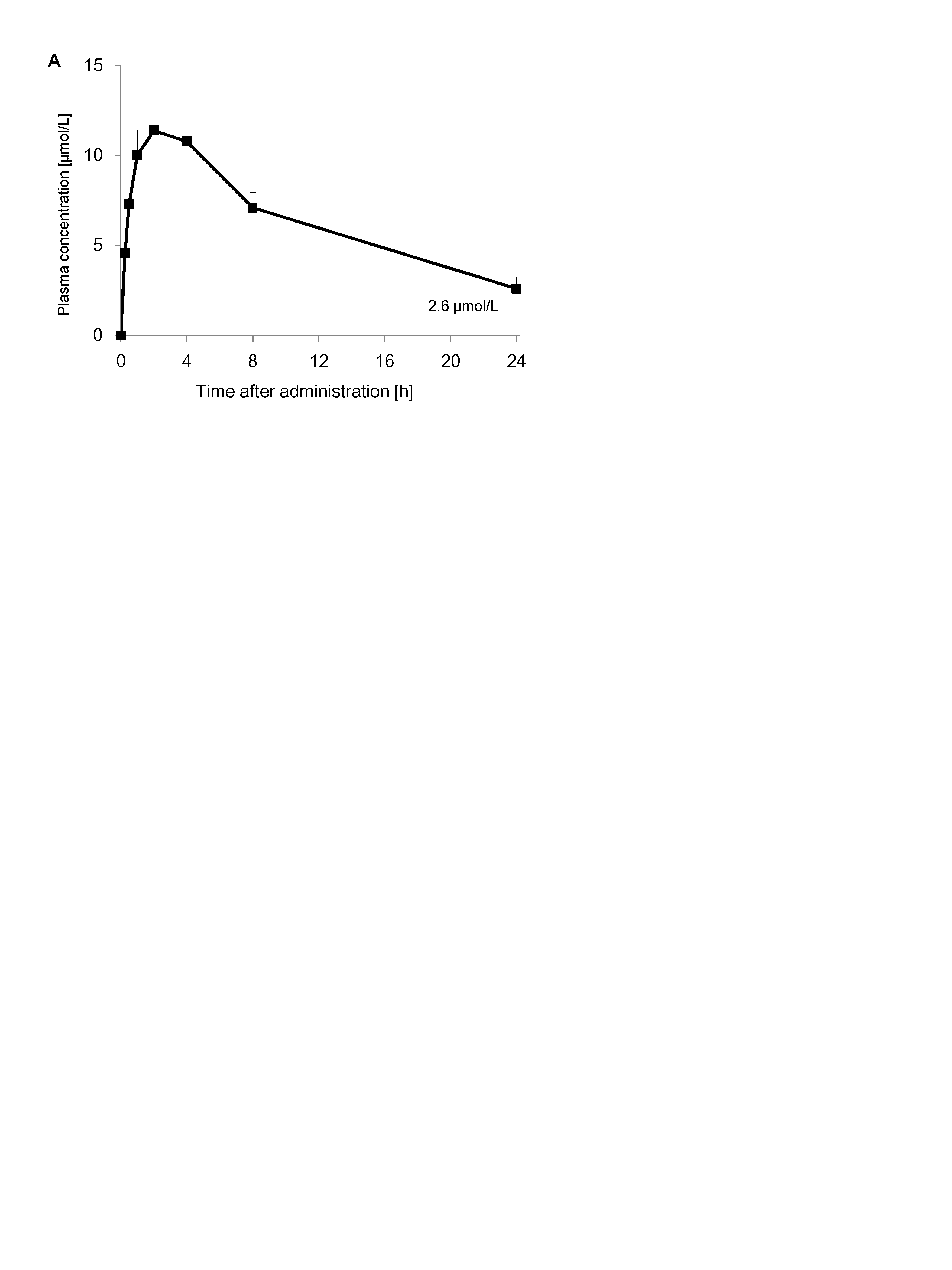

Supplement: S1 Fig — Fifty-four-week-old male C57BL/6J mice fed 45% HFD were orally administered 30 mg/kg compA suspension. Blood samples were collected at 0.25, 0.5, 1, 2, 4, 8, and 24 h after single administration. The plasma was isolated by centrifugation, and the samples were mixed with acetonitrile and centrifuged. The supernatants were diluted with an appropriate volume of 0.2% (v/v) formic acid in 10 mmol/L ammonium formate. Plasma compA levels were measured using liquid chromatography with tandem mass spectrometry (LC/MS/MS). LC/MS/MS was conducted with an API 5000 triple quadruple mass spectrometer (Applied Biosystems, Foster City, CA) coupled with a turbo ion spray interface in the positive ion mode and connected with UFLC (Shimadzu, Kyoto, Japan). Reverse-phase chromatography [mobile phase A, 0.2% (v/v) formic acid in 10 mmol/L ammonium formate; mobile phase B, acetonitrile] was used to elute and separate the different substrates with a Shim-pack XR-ODS C18 column (20 mm × 2.0 mm, 5 μm, Shimazu Co., Ltd.). Plasma concentrations at 0.25, 0.5, 1, 2, 4, 8, and 24 h after oral administration of compA at a dose of 30 mg/kg. n = 3. (TIF) [file pone.0150976.s001.tif]

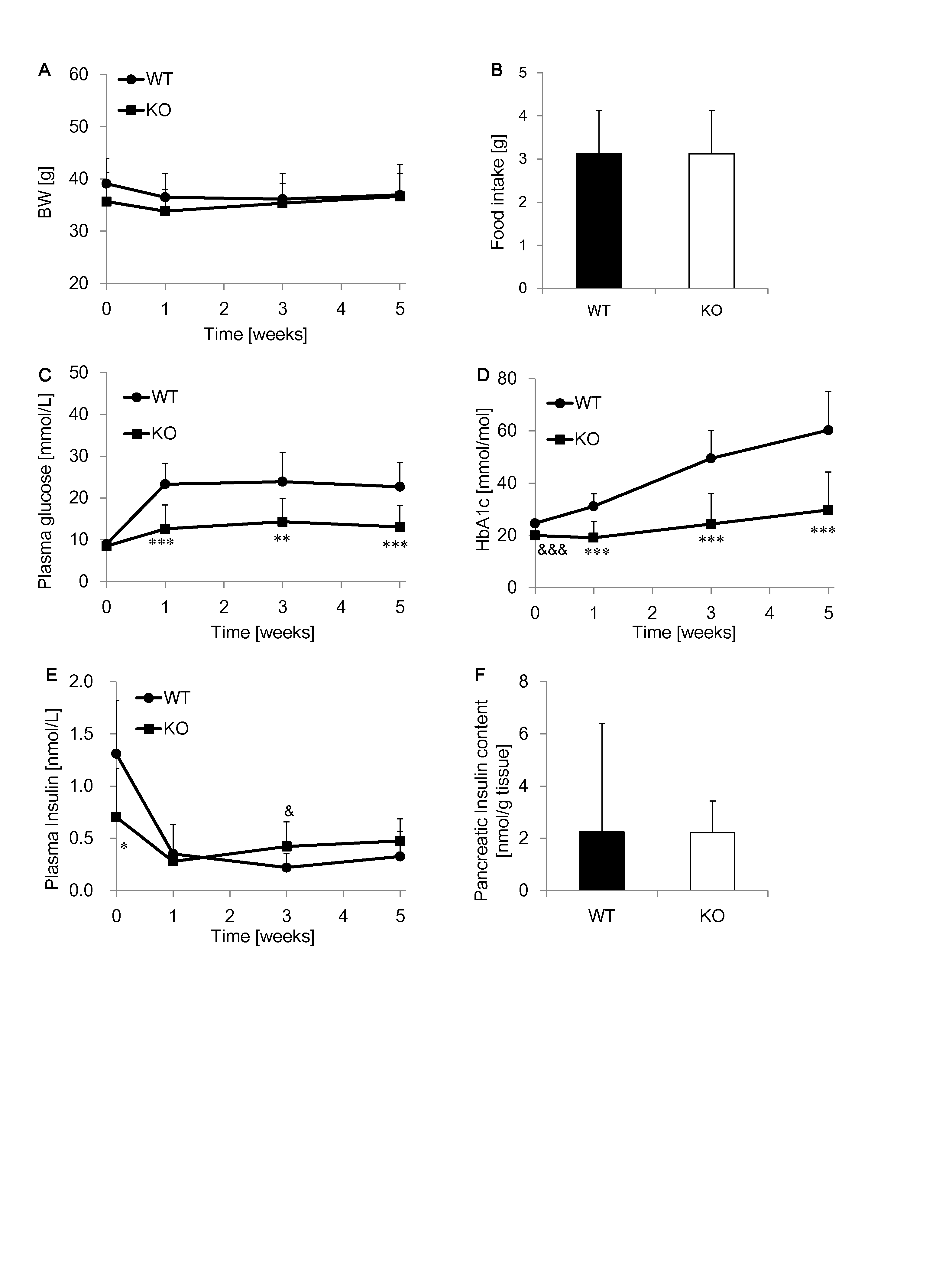

Supplement: S2 Fig — Twenty-two-week-old male MGAT2 KO mice and WT littermates were placed on 60% HFD. After 2 weeks of HFD feeding, the mice were intraperitoneally injected 90 mg/kg STZ (Sigma-Aldrich Japan, Tokyo, Japan). BW, plasma parameters, and blood GHb levels were monitored every 2 weeks. Food intake was measured 7 days after STZ administration. The pancreas was collected 6 weeks after STZ dosing under 2% isoflurane anesthesia. (A) BW. (B) Mean food intake 7 days after STZ administration. (C) Time course of changes in plasma glucose levels, (D) blood glycated hemoglobin (GHb) levels and (E) plasma insulin levels. (F) Mean pancreatic insulin levels. n = 10 (WT) or n = 11 (KO). *: P< 0.05, **: P< 0.01, ***: P< 0.001 vs. WT mice by Student’s t-test. &: P< 0.05, &&&: P< 0.001 vs. WT mice by Aspin–Welch test. (TIF) [file pone.0150976.s002.tif]

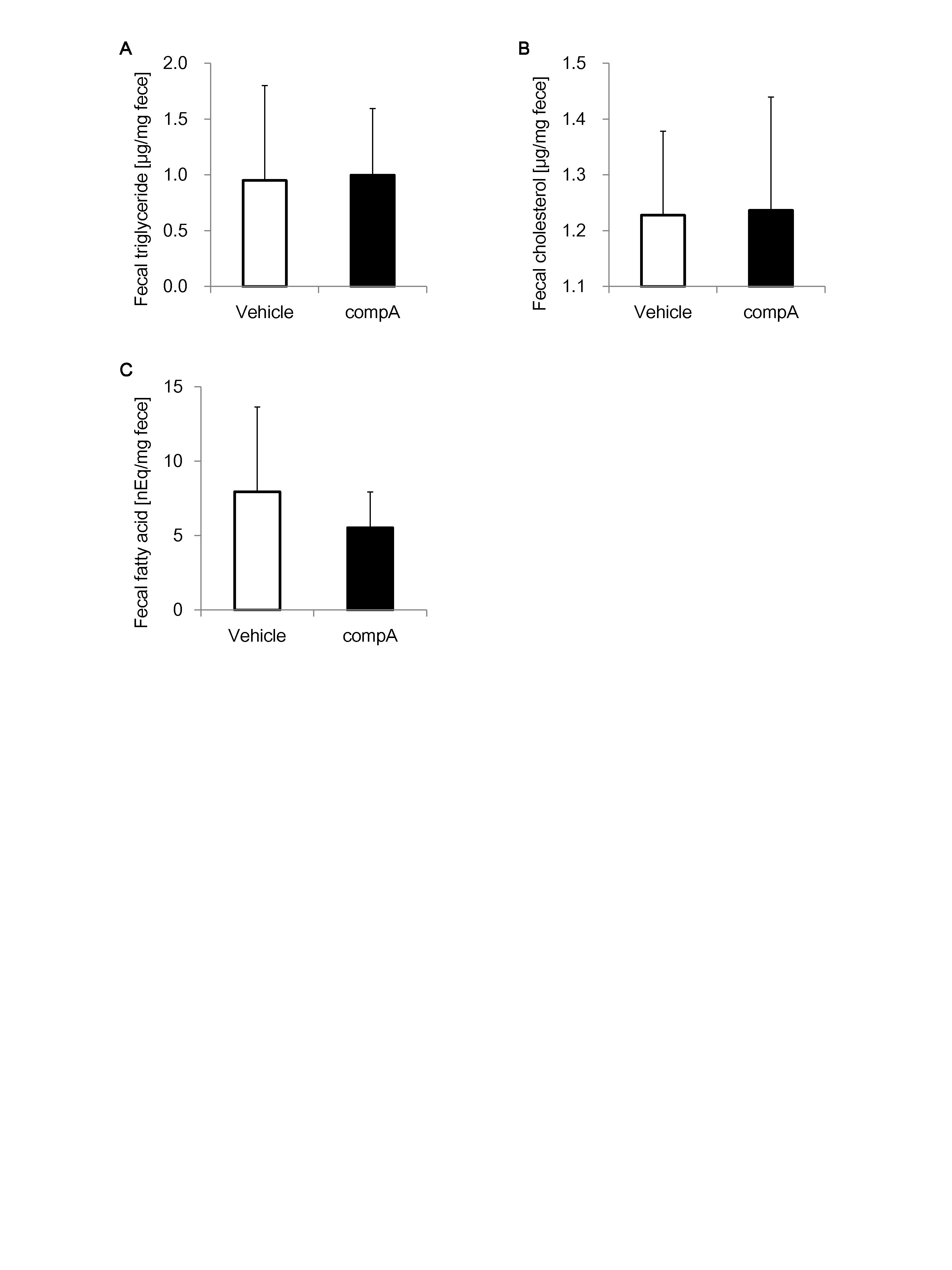

Supplement: S3 Fig — Fecal fat levels were determined by a colorimetric assay following lipid extraction with hexane/isopropanol. In brief, the fecal samples were homogenised in hexane/isopropanol mixture (3:2) using a mixer mill (Retsch, Haan, Germany) and shaken vigorously. After centrifugation, the supernatant was transferred to a tube, evaporated under nitrogen gas and resuspended in isopropanol. Sample triglyceride, cholesterol and fatty acid levels were analysed with the E-test Wako colorimetric assay kits (WAKO, Osaka, Japan). (A) Fecal triglyceride levels. (B) Fecal cholesterol levels. (C) Fecal fatty acid levels. n = 6. (TIF) [file pone.0150976.s003.tif]
